# Supplementary material for: Prevalence and predictors of obesity-related cancers among racial/ethnic groups with metabolic syndrome
Source: PLoS One. 2021 Apr 7;16(4):e0249188. doi: 10.1371/journal.pone.0249188 (PMC8026066; doi:10.1371/journal.pone.0249188)
Supplement: Attachment — Submitted filename: Response Letter for PLOS ONE.docx [file pone.0249188.s001.docx]

## Joerg Heber, PhD

# Editor in Chief

PLOS ONE

November 25, 2020

Dear Dr. Heber:

Thank you for your critical review and detailed revision suggestions for our manuscript, “Prevalence and Predictors of Obesity-Related Cancers Among Racial/Ethnic Groups with Metabolic Syndrome”. As indicated below, we have addressed each comment provided by the reviewer. Please let us know if there are any additional changes that should be made.

Additionally, we are extremely grateful for your recommendations and general willingness to help improve the quality of the paper. Thank you for the time and we look forward to hearing from you.

Sincerely,

Sincerely,

Shanada Monestime

Shanada Monestime

Assistant Professor, Hematology/Oncology-Pharmacotherapy

UNT System College of Pharmacy

2500 Camp Bowie Blvd, RES 435B

Fort Worth, Texas 76107

1. **Reviewer’s comment**: Race and ethnicity is well known to be associated with the cancer aggressiveness. For instance, the death rate in African American females is 14% higher despite 7% low incidence rate (PMID: 30558195). There seems less novelty in the study reported.
   a. **Response**: Although it is known for African Americans to have high rates of several types of cancers, our study provides novelty because we performed a regression model to further understand if after considering sociodemographic factors, did racial differences exist with obesity related cancers? Interestingly, when we divided the cancers by obesity versus non-obese cancers, African Americans were linked to having higher rates of Obesity Related Cancers and Whites were linked to having higher rates of Non-Obesity Related Cancers.
2. **Reviewer’s comment** While referring to the cardiovascular disease, authors cite relatively old reference while there are many recent evidences (PMID: 31761945, PMID: 28624099, PMID: 28421481). A careful review of cited literature is essential.
   a. **Response** The reviewer brought up a great point. Within my literature search, I have come across several recent articles between 2017-2020. The issue I am encountering is when I want to reference something from the newer articles, the statement within those articles refers back to another article (the original source) which are backdated to 2010-2014. However, I was able to update one of my references to reflect a more updated finding. Examples are below:
   b. **Response** Although reference 9 dates back from 2010, this was a review article that discussed the pathophysiology of Mets as it relates to cancer. Several new studies refers back to this article so I used it to support my statements from primary literature.
3. **Reviewer’s comment** The authors claims that the data was obtained from 1999-2014 while the reference (reference no 13) they cite is published in 1994. This need to be explained.
   a. **Response** Thank you for identifying this. I updated the references to reflect 1999-2014
4. **Reviewer’s comment** Does authors specify past vs current individuals under medication for diabetes/hypertension?
   a. **Response** Within the study population section, we included key words to indicate that survey respondents had to currently be on treatment in order for us to identify if they had hypertension and/or diabetes. For example the questions regarding medications were:
      i. Are you now taking prescribed medicine (for blood pressure)?”
      ii. “Are you now taking diabetic pills to lower blood sugar?”
      iii. Are you taking insulin now?”
   b. **Response** We did not include patients with a past medication history because we wanted to identify current status of diabetes and/or hypertension. In this section we also assessed lab values to determine diabetes and/or hypertension.
5. **Reviewer’s comment** While accessing the smoking status, does authors pay attention towards past/current smoker? It may not be relevant to classify a past smoker who at present does not smoke to classify in the third criteria (not at all a smoker).
   a. **Response** Thank you for your comment. We classified smoking status in three categories: smoking every day, some days, or not at all. Past smoking was categorized in the (not at all a smoker). Current smokers were broken down into the following two categories: smoking every day, some days. Do you mind providing clarity on the request and I am happy to update this portion of the manuscript?
6. **Reviewer’s comment** Statistical inference should be quoted in the figures (Figure 1, 2).
   a. **Response** The statistical inference for Figures 1 and 2 are now located under the image. For figure 1, our statistical analysis focused on the frequencies.
7. **Reviewer’s comment** As authors notes the observations in a range of cancer, it should be specified which cancer the obesity and metabolic syndrome related observations meet the statistical significance and what message authors like to spread through it?
   a. **Response** Thank you for this great comment. Our current funding supported a statistician to support one specific aim. Our aim was to assess if racial differences exist regarding the risks of having an obesity related cancer for patients with metabolic syndrome. Our study revealed that there is a significant risk difference by race. Therefore, we are submitting a second small grant to get funding for a statistician to assist us with determining if differences of ORC exist by gender and cancer type, while stratifying by race. Although we are unable to address cancer type into our current regression model, this manuscript serves as a foundation to demonstrate and support the next steps to identify differences by cancer types.
   b. **Response** I removed comments regarding breast and colon cancer from the discussion due to this being an observation/speculation and not supported by our statistical analysis. This redirects our manuscript to focus on our main findings. Thank you again for the great feedback.
8. **Reviewer’s comment** Which cancers authors find the gender related disparity? How does authors reconcile it with hormone status?
   a. **Response** Thank you for your excellent comment. Similar to the statement above, during our research, our primary aim focused on if racial differences exist in the risk of developing an ORC in the Mets population. Our study demonstrated that gender differences exist. Therefore, within our next small grant application we now have a foundation to support further assessing gender differences as it related to different cancer types
9. **Reviewer’s comment** As authors use only one source for the data, once the possible role of factors is identified, a careful review of literature could have conducted to justify the observations. For instance, the gender/ obesity related disparity among the observed cancers.
   a. **Response** Thank you for your comment. We revised the discussion to focus on our primary aim which was to identify if racial differences exist in the risk level of developing an ORC while confounding for several factors which prior studies did not do. Research within the Mets and ORC space by race/ethnicity is limited, but we were able to identify two main studies Steele et al and Hong et al which are listed within our discussion. that supports our findings. Within these studies they did not conduct a regression model to consider confounders so it was difficult to truly know if race/ethnicity played a role. However, how study strengthen the current findings because we performed a regression model with pertinent confounders which confirms, even with confounders in the model, racial/ethnicity differences still exist.
   b. **Response** In regards to gender differences, we were unable to find anything in Steele or Hong et al. however, we mentioned how we could use information from Chadid et al. to further analyze this data by BMI, which we plan on conducting for our second study. We needed justification as to why studying gender differences by cancer type is critical and believe our findings will serve as a foundation.
